# Fiat Lux: illuminating the cell cycle

**DOI:** 10.1038/cddiscovery.2017.42

**Published:** 2017-07-17

**Authors:** Gianmaria Liccardi, Luca L Fava

**Affiliations:** 1 The Breast Cancer Now Toby Robins Research Centre, Institute of Cancer Research, Mary-Jean Mitchell Green Building, Chester Beatty Laboratories, 237 Fulham Road, London SW3 6JB, UK; 2 Medical University of Innsbruck, Biocenter, Division of Developmental Immunology, Innsbruck A-6020, Austria

The ordered series of events leading to the exact duplication of a cell is known as the cell division cycle (or simply cell cycle). Its most critical phases are DNA synthesis (S-phase), when the genome is duplicated, and cell division or mitosis (M-phase), during which condensed chromosomes are equally distributed into two daughter cells. These two phases are separated by ‘gap’ phases (Gap1 or G1 before S-phase and Gap2 or G2 before M-phase). During G1, cells make a critical decision about growth *versus* quiescence and check their DNA before entering S-phase. During G2, cells enter a phase of rapid growth and protein synthesis while preparing to divide.^1^ Together these four stages encompass the life cycle of most cells in many living organisms and allow the dynamic interaction of every signaling pathway known. This process is highly heterogeneous with regard to cycling times (varying from 20 min to many hours and in some cases days), p53 dependency and, most importantly, the convergence of many different biochemical events that allow transition from one phase to another. The study of such complex process is critical for cell biology, and live-cell imaging allows the visualisation of all the dynamic changes taking place. This provides many more insights into the processes that lead to the activation of one signaling pathway over another as compared to single snapshots provided by imaging fixed cells or analysis of the DNA content or protein extracts.

Historically, the study of M-phase greatly profited of live-cell imaging that allowed specific visualisation of a finely regulated sequence of events in real time, affording an otherwise impossible mechanistic understanding of the mitotic process.^2^ In this perspective, the interphase remained for a long time defined by exclusion, as its internal transitions have long been not resolvable in live-cell imaging. Thus, the study of interphase was confined to ‘snapshot’ approaches in which cell cycle phase distribution can be assessed on fixed specimens, such as with BrdU incorporation into chromatin as a reporter for S-phase activity. The use of genetically encoded fluorescent proteins represented a breakthrough in the resolvability of cell cycle phases in living specimens, and this allowed not only to label cellular structures that display a dynamic behavior in the cell cycle, such as chromatin, but also to report with high precision on the cycle-regulated protein degradation by the ubiquitin−proteasome system (UPS).^3,4^ Relying on the ability of the UPS to degrade fluorescent proteins fused to cell cycle-regulated proteins, a first fluorescent ubiquitination-based cell cycle indicator (FUCCI) was developed almost 10 years ago.^5^ The FUCCI system exploits the antiphase oscillatory behavior of two key regulators of DNA replication, CDT1 and Geminin. While the origin of replication licensing factor CDT1 accumulates in G1 and then vanishes upon S-phase entry, Geminin levels start rising during S-phase and are maintained till late M-phase, allowing inhibition of Cdt1 and therefore inhibiting DNA re-replication. The alternating expression of these two proteins depends on the sequential activation of the E3 ubiquitin ligases SCF^Skp2^ (a Skp1−cullin-1−F-box complex associated to Skp2 as the F-box protein) and the anaphase-promoting complex/cyclosome associated to its co-activator Cdh1 (APC/C^Cdh1^), which target CDT1 and Geminin for degradation, respectively (Figure 1a). As the ectopic expression of both CDT1 and Geminin perturbs the cell division cycle, the FUCCI system relied on the minimal amino-acid sequence (annotated with lower script next to the protein of interest) known to suffice for conferring regulated degradation to the fusion protein, but *per se* insufficient to alter cell cycle dynamics (Figure 1b). The FUCCI system has allowed resolving the cell cycle distribution in living specimens, contributing to (i) understanding its coordination with other processes such as tissue and organ morphogenesis during development,^5,6^ (ii) assessing the propensity of stem cells to differentiate in relation to the cell cycle distribution,^7^ (iii) enriching for cells in certain cell cycle windows by flow cytometry independently of their DNA content,^8^ and (iv) studying the cell cycle perturbations induced by chemotherapeutic drugs,^9^ to name a few applications.

Despite having revolutionized the live-cell imaging of cell cycle transitions, the FUCCI system presents a crucial limitation. It essentially reveals if cells are within any of the three proliferative phases of the cell cycle (S-G2-M) without, however, discerning among these three. Exact live visualisation of all the different phases has been a long-awaited biological accomplishment. This is mainly because it is becoming more and more clear that specific modulation of individual cell cycle phase affects cell movement, development, DNA repair, cell death and cancer biology, but, most importantly, cancer therapy and its response.

The paper by Bajar *et al.*^10^ provides a modification of the FUCCI system proposing the FUCCI4, which allows specific discrimination between each of the cell cycle phases (Figure 1a). The innovation of this method consists in the adoption of a new fluorescent protein to visualise chromatin condensation during M-phase and an intensiometric reporter that specifically detects the transition from S to G2.

The intensiometric reporter is based on the human stem-cell loop binding protein (SLBP), an RNA binding protein that is degraded at the end of S-phase.^11^ The authors find that the fusion of m-Turquoise2 with the amino-acid sequence 18−126 of the SLBP protein (SLBP_18−126_) is sufficient for the proteasome machinery to degrade mTurquoise2 signal at the end of S-phase, rendering possible the clear definition between S and G2.

While the introduction of a mitotic specific label perhaps was not, *per se,* a novelty, the authors elegantly discover a new fluorescent protein: mMaroon1. This is then fused to Histone H1 (H1) to detect chromatin condensation during mitosis. mMaroon1 contains 26 mutations from the original fluorescent protein mNeptune2 far-RFP and is threefold brighter than tag RFP657. The real advantage, besides the undetectable photobleaching, is that mMaroon1 emission starts at a longer wavelength compared to other far-RFPs. This means that orthogonal fluorescent protein detection up to 590 nm does not detect mMaroon1, allowing the possibility of labelling two proteins within the orange to far-red spectra and therefore simultaneous four-channel imaging. Hence, live-cell imaging with Turquoise2, clover, mKO2 and mMaroon1 (cyan, green, orange and far-red) allows orthogonal imaging without any detectable bleedthrough.

The FUCCI4 represents therefore a true scientific ‘Fiat Lux’ (‘Let there be light’) compared to the rather darker bi-fluorescent ancestor FUCCI (Figure 1). The system utilises specifically m-Turquoise−SLBP_18−126_, H1.0 Maroon1, Clover-Geminin_1−110_ and mKO2-Cdtl_30−120_. G1−S transition is marked by gradual appearance of Clover-Geminin_1−110_ while m-Turquoise−SLBP_18−126_ persists through the S-phase. End of S-phase and beginning of G2 is marked by loss of m-Turquoise−SLBP_18−126_ and persistence of Clover-Geminin_1−110_. M-phase is marked by nuclear envelope breakdown and chromosome condensation, visualised by H1.0-Maroon1 (while Clover-Geminin_1-110_ is persisting). Finally, loss of Clover-Geminin_1−110_ and H1.0 Marroon1 and appearance of mKO2-Cdtl_30−120_ and m-Turquoise−SLBP_18-126_ mark the beginning of G1 (Figure 1).

Some considerations are however important. While H1.0 Maroon1 markers can track cells during cytokinesis before the G1 label become visible, which is a novelty in the visualisation of cytokinesis outside of G1 interphase, such application is not always required. Mitosis can be scored by other means in living cells, e.g. using phase or differential interference contrast imaging or by utilising cell permeable dyes such as SiR-Hoechst that emit in the far-red region.^12^ The latter also allows orthogonal imaging with the remaining three dyes, reducing therefore the number of transgenes to integrate. Despite the potency of such system, not all cell lines (primary or transformed) are easily manipulated, in particular those derived by primary tumours. Hence, the exact cellular setting and the extensibility of this technique still await experimental validation. The greatest advantage that the FUCCI4 presents is certainly the ability to distinguish between G2 and S during live-cell imaging. Furthermore, the implications of this technique extend to many different biological fields: (i) screening of drugs that manipulate specific stages of the cell cycle, (ii) study of oncogene-driven replication stress, (iii) molecular characterisation of cell cycle phase transition, (iii) understanding the resistance to nucleoside analogues utilised to treat many types of cancer, (iv) study of the effects on cell cycle by different developmental signals, cytokine production, cancer, modulation of microenvironment, cell death, DNA damage repair and cell survival.

## Figures and Tables

**Figure 1:**
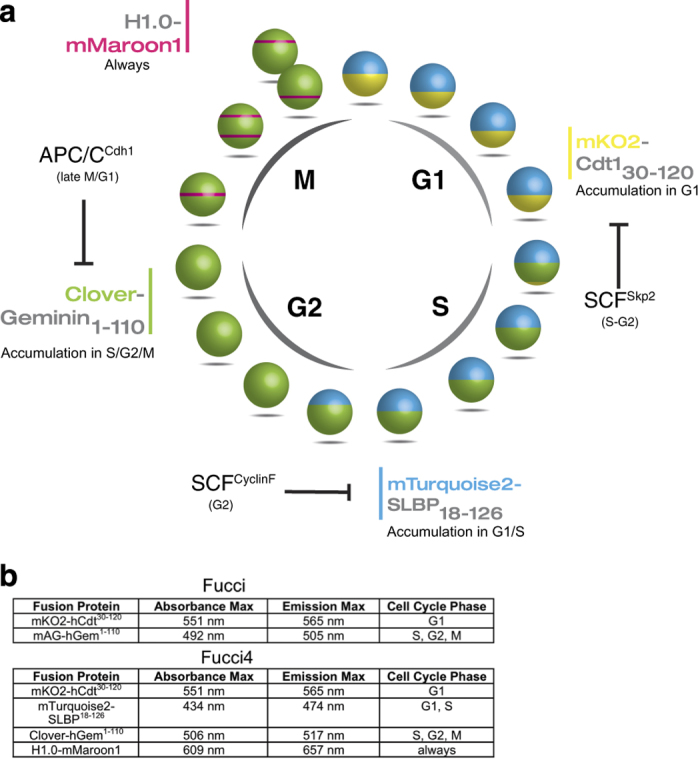
Graphic representation of the FUCCI4 system: adaptation from Bajar *et al.*^10^ (**a**) Cell cycle scheme showing the individual cell cycle phase marker, the attached fluorophore and the minimal amino-acid sequence sufficient for protein degradation. The diagram also shows the UPS responsible for the specific degradation of the fusion proteins. (**b**) The table shows the difference between FUCCI and FUCCI4 highlighting the absorbance and the emission of each FP.
